# Ectrodactyly-lobster claw deformity

**DOI:** 10.11604/pamj.2021.40.80.26009

**Published:** 2021-10-06

**Authors:** Sarath Kumar Udaya Kumar, Krishna Prasanth Baalan

**Affiliations:** 1 Department of Community Medicine, Sree Balaji Medical College and Hospital, Bharath University, Chennai, India

**Keywords:** Deformity, children, congenital

## Image in medicine

Lobster-claw deformity, also called ectrodactyly is an uncommon congenital presentation occurring in the hand due to longitudinal failure of development of second, third or fourth ray. It occurs in 1 to 4 newborns in 100,000 live births. It occurs mostly due to consanguineous marriage but can also occur in the non-consanguineous marriage. This deformity is usually associated with ectodermal defects, mental retardation, deafness, orofacial clefting and tibial aplasia. A 6-year-old boy was brought to the Department of Orthopedics in a Private Medical Hospital, Chennai, with deformity of left hand since birth. The affected hand had a wide median cleft and, there was deformity of flexion of the ring and index finger with absence of middle finger. The other hand and both feet were normal. On radio-imaging, left hand showed transversely oriented bone between third and fourth metacarpal and phalanges of middle finger were absent except for rudimentary proximal phalanx. No other congenital defects were present other than this. The child was born to non-consanguineous parents with no significant family history. The treatment for this anomaly is complete surgical excision of transverse bone with partial excision of the third metacarpal and apposition of the second and fourth metacarpal by absorbable sutures which leads to a smaller cleft. If required, flexion contracture of ring and index finger will be corrected.

**Figure 1:**
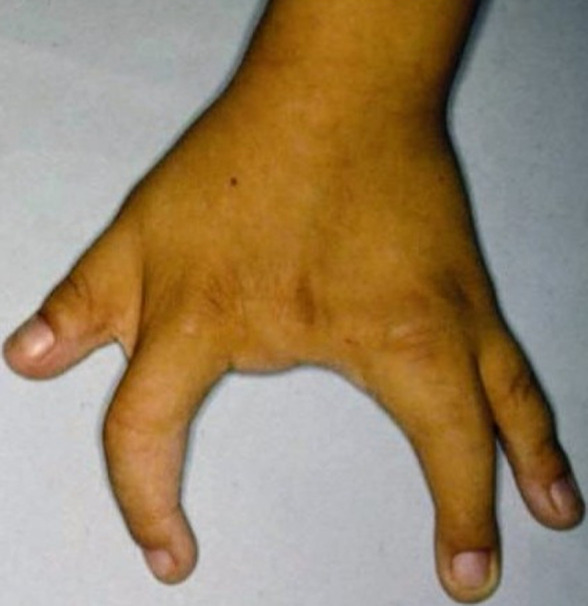
lobster claw deformity

